# AI in Esophageal Motility Disorders: Systematic Review of High-Resolution Manometry Studies

**DOI:** 10.2196/85223

**Published:** 2025-11-27

**Authors:** Eun Jeong Gong, Chang Seok Bang, Jae Jun Lee, Gwang Ho Baik

**Affiliations:** 1 Department of Internal Medicine, Hallym University College of Medicine Chuncheon Republic of Korea; 2 Institute for Liver and Digestive Diseases Hallym University Chuncheon Republic of Korea; 3 Institute of New Frontier Research Hallym University College of Medicine Chuncheon Republic of Korea; 4 Department of Internal Medicine Hallym University College of Medicine Chuncheon, Gangwon Republic of Korea; 5 Department of Anesthesiology and Pain Medicine Hallym University College of Medicine Chuncheon Republic of Korea

**Keywords:** manometry, artificial intelligence, machine learning, deep learning, motility, high-resolution manometry, esophageal motility disorders, Chicago Classification, systematic review

## Abstract

**Background:**

High-resolution esophageal manometry (HRM) is essential for diagnosing esophageal motility disorders, affecting 10%-15% of patients with dysphagia. Current interpretation via the Chicago Classification remains challenging, with interobserver variability reaching 30%-40% even among experts. Artificial intelligence (AI) has emerged as a transformative tool to automate HRM interpretation.

**Objective:**

We aimed to evaluate current AI HRM applications and assess diagnostic accuracy, methodological approaches, clinical validation, implementation barriers, and real-world implications for gastroenterology practice.

**Methods:**

We searched PubMed/MEDLINE, Embase, Cochrane Library, and Web of Science through November 2025, for studies using AI or machine learning to interpret esophageal HRM. Eligible studies included original research evaluating such interpretation in adults with esophageal symptoms, published in English. We excluded case reports, reviews, abstracts, and studies without outcomes. Data on AI model tasks and diagnostic outcomes were extracted. Primary outcomes included diagnostic accuracy metrics, secondary outcomes encompassing external validation performance, real-time processing capabilities, and comparison with expert interpretation. Two reviewers independently screened studies and extracted data. Study quality was appraised using QUADAS-2 (Quality Assessment of Diagnostic Accuracy Studies-2) criteria. Given the substantial heterogeneity, we performed qualitative narrative synthesis rather than quantitative meta-analysis.

**Results:**

Seventeen studies encompassing 4588 patients demonstrated progressive AI evolution across 3 phases. Early studies (2013-2016, n=4) using traditional machine learning achieved 86.5%-94% accuracy for parameter extraction. Deep learning era (2018-2022, n=8) achieved breakthrough performance: 97% (95% CI 95.7%-98.3%) accuracy for integrated relaxation pressure classification, 91.32 (95% CI 87.0%-94.5%) for motility tracing, and 86% for complete Chicago Classification automation. Recent multimodal approaches (2023-2024, n=5) incorporating acoustic analysis and fuzzy logic achieved 83%-95% accuracy while reducing interpretation time from 15-20 to <2 minutes. AI systems demonstrated superior consistency with 0 intraobserver variability compared to 15%-30% among human experts. However, critical gaps emerged: 0% (0/17) of studies performed external validation, 82% (14/17) showed unclear patient selection bias, and none obtained regulatory approval. QUADAS-2 assessment identified low risk of bias in 65% (11/17) of studies for the index test domain but high concern in 100% for applicability due to lack of real-world testing.

**Conclusions:**

This review demonstrates AI’s transformative potential for HRM interpretation, with diagnostic accuracies reaching 97%. Real-world implications are significant, promising to enable standardized diagnostics across institutions, address the critical shortage of motility experts affecting 70% of global health care systems, and reduce health care costs by 20%-30% through an 85%-90% reduction in interpretation time and decreased repeat procedures. Beyond synthesizing existing evidence, this review brings new knowledge to the field through 3 key contributions: mapping the evolutionary trajectory from rule-based to deep learning systems, quantifying AI’s superior reproducibility compared to human experts, and revealing the critical disconnect between algorithmic performance and clinical translation. Future priorities include multicenter validation trials and regulatory pathway development.

**Trial Registration:**

PROSPERO CRD420251154237; https://www.crd.york.ac.uk/PROSPERO/view/CRD420251154237

## Introduction

The diagnosis and classification of esophageal motility disorders have undergone evolution since the introduction of high-resolution esophageal manometry (HRM) in the early 2000s [1]. This technological advancement, characterized by closely spaced pressure sensors providing spatiotemporal pressure topography displays, has altered our understanding of esophageal physiology and pathophysiology [1,2]. The subsequent development and iterative refinement of the Chicago Classification, now in its fourth version, has established a standardized framework for HRM interpretation that has become the global standard for esophageal motility assessment [3,4]. Despite these advances, significant challenges persist in clinical practice, including substantial interobserver variability even among expert interpreters, time-intensive analysis requirements, and the need for extensive training to achieve competency in HRM interpretation [5,6].

In recent years, interest in applying artificial intelligence (AI) to medical data has surged [7,8]. AI in medicine encompasses methods ranging from classical statistical models to advanced deep learning and even generative models. These approaches can rapidly analyze large datasets and automatically extract complex features, making them well-suited to assist in health care data interpretation [9]. Gastroenterology has seen rapid exploration of AI for endoscopic image analysis, pathology slide interpretation, and other tasks [10]. Recent comprehensive reviews have demonstrated AI’s expanding role across gastroenterological applications, from polyp detection to diagnostic decision support systems, with particular promise in image-based diagnostics [11]. Large language models have also emerged as potential tools for clinical documentation and patient education in gastroenterology, though their role in technical interpretation remains under investigation [12]. Within the field of neurogastroenterology and motility, AI technologies offer particularly compelling advantages given the pattern-based nature of HRM interpretation and the quantitative parameters inherent to manometric analysis. Machine learning algorithms excel at pattern recognition tasks, potentially surpassing human capabilities in identifying subtle abnormalities and maintaining consistent diagnostic criteria application [13,14]. Furthermore, AI systems can process vast quantities of data instantaneously, enabling real-time interpretation that could transform clinical workflow efficiency [7,10]. Recent reviews have examined AI applications in general gastroenterology [7-10]. However, a focused analysis of HRM-specific applications remains lacking.

The evolution of AI methodologies in medical imaging and signal processing has particular relevance to HRM analysis [15]. Early applications relied on traditional machine learning approaches such as support vector machines and random forests, which required manual feature extraction and engineering [10,16]. These methods, while showing promise, were limited by their dependence on predefined features and inability to capture complex spatiotemporal patterns inherent to esophageal pressure topography. The advent of deep learning, particularly convolutional neural networks (CNNs), has revolutionized medical image analysis by enabling automatic feature learning directly from raw data [10,17]. For HRM, this capability allows AI systems to identify novel patterns and relationships that may not be apparent to human observers or captured by traditional metrics. Recent systematic assessments of AI tools in esophageal dysmotility diagnosis have documented the progression from basic automation of landmark identification to sophisticated deep learning models capable of comprehensive Chicago Classification diagnosis [18]. Contemporary applications now encompass not only HRM but also impedance-pH monitoring, demonstrating the broadening scope of AI in esophageal diagnostics [19].

Recent technological advances have further expanded the potential applications of AI in esophageal motility assessment. The integration of complementary diagnostic modalities, such as Functional Luminal Imaging Probe (FLIP) technology and high-resolution impedance manometry, provides multidimensional data that can enhance diagnostic accuracy [19]. AI platforms have demonstrated 89% accuracy in automated interpretation of FLIP Panometry studies, validating the feasibility of automated esophageal motility classification during endoscopy [20]. AI systems are uniquely positioned to synthesize these complex, multimodal datasets, potentially revealing pathophysiological insights that single-modality assessment cannot provide [11]. Moreover, the development of cloud-based computing infrastructure and edge computing capabilities enables the deployment of sophisticated AI models in diverse clinical settings, from tertiary referral centers to community practices [21,22]. The emergence of generative artificial intelligence and large language model–assisted development has further accelerated model creation, with recent studies demonstrating the successful implementation of Gemini-assisted (Google LLC) deep learning for automated HRM diagnosis, achieving high diagnostic precision across multiple motility disorder categories [23].

Despite these promising developments, no comprehensive systematic review has evaluated the full spectrum of AI applications in HRM interpretation or assessed their methodological quality. Therefore, this systematic review aims to (1) systematically evaluate current AI applications in HRM interpretation, (2) assess diagnostic accuracy across different AI methodologies, (3) evaluate methodological quality, and (4) identify barriers to clinical implementation and future research priorities.

## Methods

### Study Design

The protocol was registered in PROSPERO (International Prospective Register of Systematic Review; CRD420251154237) before initiating the search. This systematic review followed the PRISMA (Preferred Reporting Items for Systematic Reviews and Meta-Analyses) 2020 reporting guidelines [24] (Multimedia Appendix 1), PRISMA-Diagnostic Test Accuracy (Multimedia Appendix 2) checklist [25], and PRISMA-S (Preferred Reporting Items for Systematic Reviews and Meta-Analyses-Search, an extension to the PRISMA statement for reporting literature searches in systematic reviews; Multimedia Appendix 3) checklist [26].

### Database and Searching Strategy

We searched PubMed/MEDLINE, Embase, Cochrane Library, and Web of Science through September 2025, for studies using AI or machine learning to interpret esophageal HRM. Search strategies incorporated keywords and indexed terms, including (“artificial intelligence” OR “machine learning” OR “deep learning” OR “neural network” OR “computer-aided diagnosis”) AND (“high-resolution manometry” OR “HRM” OR “esophageal manometry” OR “esophageal motility” OR “Chicago Classification”; Textbox 1). Gray literature sources were searched to reduce publication bias.

**Searching strategy to find the relevant papers. Comprehensive search strategies were used to identify studies on artificial intelligence (AI) applications in HRM across 4 databases. Search strategies used MeSH (Medical Subject Headings) and Emtree keywords searched as free-text terms in titles and abstracts covering: (1) AI/machine learning concepts, (2) esophageal motility disorders and gastrointestinal motility, and (3) HRM/esophageal physiologic testing. Optimizing search sensitivity: we empirically tested both approaches (eg, “Gastrointestinal motility”[tiab] vs “Gastrointestinal motility”[Mesh]) and found that searching MeSH keywords as free-text in (title and abstract [tiab]) yielded more comprehensive results. This captures papers using these established terms that may not yet be formally indexed with the corresponding MeSH headings, or where these concepts appear in titles or abstracts but are not assigned as subject headings. Searches were conducted from database inception through September 24, 2025 (initial search) and updated October 27, 2025, and verified for reproducibility on November 6, 2025, with no language restrictions. The table displays exact search syntax for MEDLINE via PubMed, Embase via OVID, Cochrane Library via Wiley, and Web of Science Core Collection, along with the number of records retrieved from each source (lang: language; ab.ti.kw: abstract, title, and keyword; and ab: abstract).**

**Database: MEDLINE (through PubMed)**

#1 “artificial intelligence”[tiab] OR “machine learning”[tiab] OR “deep learning”[tiab] OR “neural network”[tiab] OR “computer-aided diagnosis”[tiab]: 345034

#2 “high-resolution manometry”[tiab] OR “HRM”[tiab] OR “esophageal manometry”[tiab] OR “esophageal motility”[tiab] OR “Chicago Classification”[tiab] OR “Gastrointestinal motility”[tiab]: 15092

#3 #1 AND #2: 116

#4 #3 AND English[Lang]: 114

**Database: Embase-OVID**

#1 'artificial intelligence':ab,ti,kw OR 'machine learning':ab,ti,kw OR 'deep learning':ab,ti,kw OR 'neural network':ab,ti,kw OR 'computer-aided diagnosis':ab,ti,kw: 173049

#2 'high-resolution manometry':ab,ti,kw OR 'HRM':ab,ti,kw OR 'esophageal manometry':ab,ti,kw OR 'esophageal motility':ab,ti,kw OR 'Chicago Classification':ab,ti,kw OR 'Gastrointestinal motility ':ab,ti,kw: 38254

#3 #1 AND #2: 73

#4 #3 AND ([article]/lim OR [article in press]/lim OR [review]/lim) AND [English]/lim: 39

**Database: Cochrane Library (Through Wiley)**

#1 'artificial intelligence':ab,ti,kw OR 'machine learning':ab,ti,kw OR 'deep learning':ab,ti,kw OR 'neural network':ab,ti,kw OR 'computer-aided diagnosis':ab,ti,kw: 11482

#2 'high-resolution manometry':ab,ti,kw OR 'HRM':ab,ti,kw OR 'esophageal manometry':ab,ti,kw OR 'esophageal motility':ab,ti,kw OR 'Chicago Classification':ab,ti,kw OR ‘Gastrointestinal motility’:ab,ti,kw: 4636

#3 #1 AND #2: 36

**Database: Web of Science**

#1 ab=(“artificial intelligence” OR “machine learning” OR “deep learning” OR “neural network” OR “computer-aided diagnosis”): 645285

#2 ab=(“high-resolution manometry” OR “HRM” OR “esophageal manometry” OR “esophageal motility” OR “Chicago Classification” OR ‘Gastrointestinal motility’): 9769

#3 #1 AND #2: 138

Additional information sources were systematically searched to identify gray literature and unpublished studies. We searched the medRxiv preprint server [27] using the same search terms to identify studies not yet formally published (advanced searching tab). ClinicalTrials.gov [28] was searched to identify ongoing or completed trials that may not have been published. Reference lists of all included studies and relevant systematic reviews were manually screened to identify additional eligible studies. No citation reference searches were performed using citation databases.

The search strategy was peer reviewed by information scientists who have extensive expertise in systematic review methodology and database search strategies.

The results from all database searches were exported and deduplicated using EndNote X20 (Clarivate Analytics, 2020). Automated deduplication was performed using EndNote’s duplicate identification algorithm, followed by manual review to identify and remove any remaining duplicates based on title, author, year, and journal. Two reviewers (CSB and EJG) independently screened studies, and discrepancies were resolved by discussion (Multimedia Appendix 4).

### Inclusion and Exclusion Criteria

We included both prospective and retrospective studies that applied an AI-based algorithm to HRM measurements for diagnosing or classifying esophageal motility disorders (eg, achalasia subtypes, esophagogastric junction outflow obstruction, distal esophageal spasm, hypercontractile esophagus, ineffective motility, etc). We excluded nonhuman studies, conference abstracts without full text, studies focusing on anorectal manometry, and studies on other modalities (such as FLIP or pH-impedance) unless they directly involved HRM data integration.

The detailed inclusion criteria are as follows: (1) original research applying AI, machine learning, or deep learning techniques to HRM data; (2) evaluation of diagnostic accuracy, classification performance, or clinical outcomes; (3) inclusion of human participants or HRM studies; and (4) provision of quantitative performance metrics. The exclusion criteria are as follows: (1) review papers, editorials, or case reports without original data; (2) used only conventional manometry without high-resolution capabilities; (3) applied AI exclusively to other esophageal diagnostic modalities without HRM integration; and (4) lacked sufficient methodological detail for quality assessment.

### Data Extraction

Two independent reviewers (CSB and EJG) systematically extracted data using a standardized, prepiloted form. Extracted variables included: study characteristics (authors, year, country, and design), patient demographics (sample size, age, and sex distribution), HRM technical specifications (equipment, protocol, and Chicago Classification version), AI methodology (algorithm type, architecture, and training approach), dataset characteristics (size, split ratios, and validation method), performance metrics (sensitivity, specificity, accuracy, and area under the receiver operating characteristic curve [AUROC]), clinical outcomes when available, and implementation considerations. Discrepancies were resolved through consensus or third reviewer (GHB) arbitration. Authors were contacted for missing or unclear data, with a maximum of 3 contact attempts over 4 weeks.

### Study Outcomes

Primary outcome measures included diagnostic accuracy metrics for AI systems compared to expert interpretation as the reference standard. Sensitivity, specificity, positive and negative predictive values, and accuracy were calculated when raw data were available. For studies reporting only AUROC values, these were extracted directly. Meta-analysis was planned if sufficient homogeneity existed across studies; however, due to significant heterogeneity in AI approaches, patient populations, and outcome definitions, a narrative synthesis was performed.

Secondary outcomes included: external validation performance compared to internal validation, processing time for automated interpretation, comparison with trainee interpretation, interrater reliability metrics, and clinical outcomes when reported. Subgroup analyses examined performance differences by: AI methodology (traditional machine learning vs deep learning), disorder category according to the Chicago Classification, validation approach (internal vs external), and year of publication to assess temporal trends.

### Quality Assessment

We assessed the methodological quality and risk of bias of each included study using the QUADAS-2 (Quality Assessment of Diagnostic Accuracy Studies-2) tool. This tool evaluates risk of bias in 4 domains: patient selection, index test, reference standard, and flow and timing. For each domain, we judged the risk of bias as low, high, or unclear based on the information reported in the study, and we also noted any concerns regarding applicability to the review question [29]. Two reviewers (CSB and EJG) performed the QUADAS-2 assessments independently, with disagreements resolved through discussion.

## Results

### Study Selection and Inclusion

**Figure 1:**
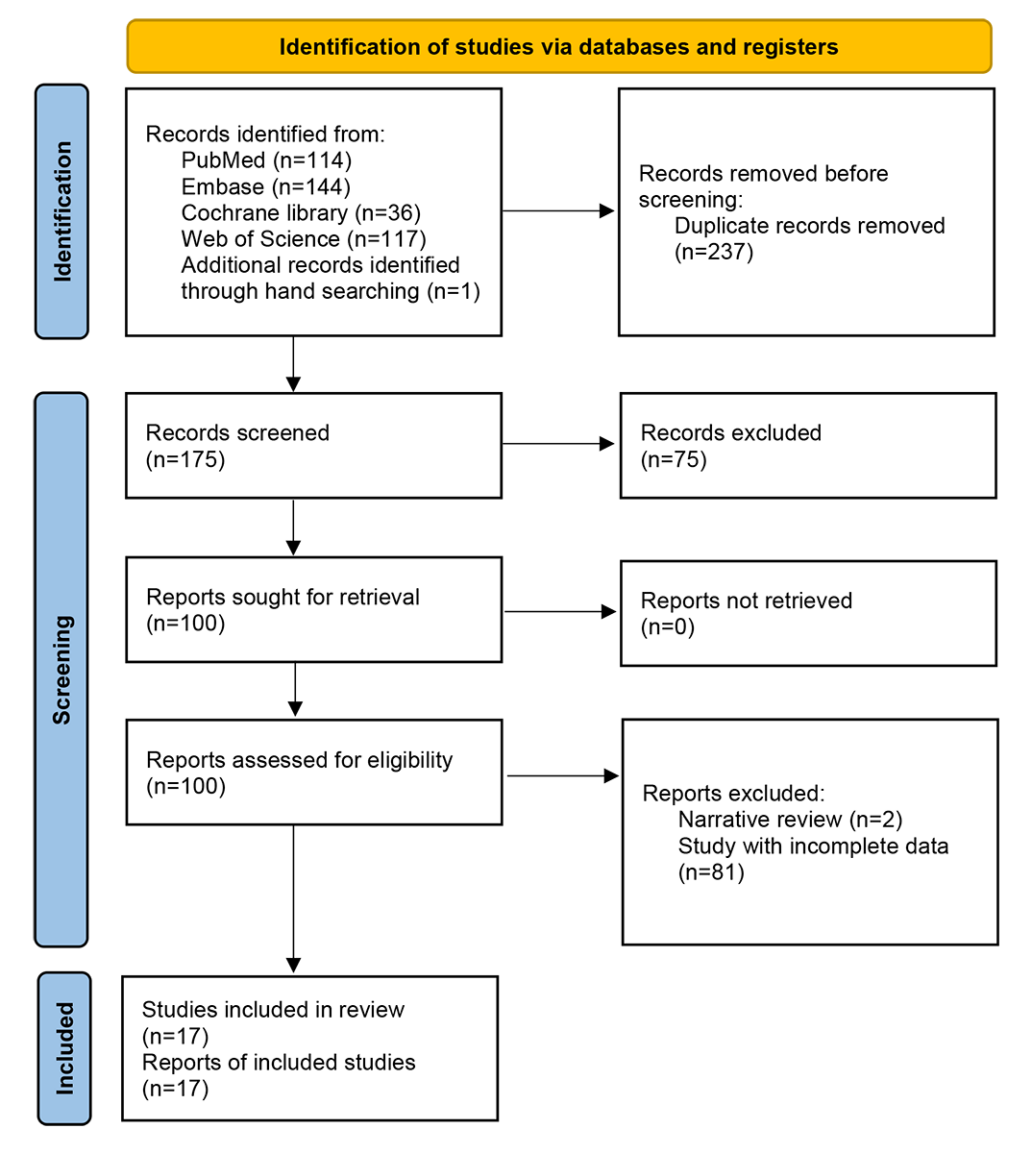
Study selection flow.

Literature search yielded 411 studies from databases and 1 additional record from manual screening. After removing duplicates, 175 studies remained. Following title and abstract screening, 100 full-text papers were assessed for eligibility. Of these, 83 were excluded. Ultimately, 17 studies met inclusion criteria (Figure 1).

Figure 1 is the PRISMA flow diagram for systematic review of AI applications in HRM (2013-2025). Literature search across PubMed/MEDLINE, Embase, Cochrane Library, and Web of Science (database inception through November 2025) identified studies applying AI, machine learning, or deep learning techniques to interpret HRM for diagnosis of esophageal motility disorders. The diagram illustrates the screening process.

### Study Characteristics

Studies were published between 2013-2025, with 82% (14/17) of the studies published in 2020 or later. The studies with clearly documented patient numbers included: Hoffman et al [30], with 30 participants with dysphagia, Rohof et al [31], 50 patients with gastroesophageal reflux disease, Jungheim et al [32] with 15 healthy volunteers, Kou et al [33] with 2161 HRM cases, Kou et al [34] study with 1741 HRM cases, Wang et al [35] with 229 esophageal motility cases from 229 individuals, Surdea-Blaga et al [36] with 192 HRM studies (patients), Rafieivand et al [37] with 67 patients, Zifan et al [38] with 60 patients, and Lankarani et al [39] with 43 patients. The total confirmed patient count from studies with explicit numbers was at least 4588 patients, though several studies did not report exact patient numbers. Publication years ranged from 2013 to 2025, with 82% (14/17) published after 2020, reflecting the recent emergence of this field. Study designs were predominantly retrospective cohort studies (n=15, 88%), with 2 methodological development studies (n=2, 12%; Rohof et al [31] and Kou et al [33]). No prospective validation studies were identified. All studies used the Chicago Classification as the reference standard, with varying versions used across studies (Table 1).

**Table 1:** Summary of the included studies^a^.

Study and year	Country	Sample size	AI^b^ method	Study aims	Performance	Validation	Chicago classification
Hoffman et al, 2013 [30]	United States	30 participants 335 swallows Dysphagia 19 men and 11 women mean age: 68.0 (SD 11.8) years	Multilayer perceptron artificial neural network	Pharyngeal analysis 7 MBSImP^c^ components	Accuracy: 91% AUROC^d^: 0.90-0.98	Internal validation only	Unspecified
Rohof et al, 2014 [31]	Australia	50 patients GERD^e^ 33 men and 17 women Mean age 52 (SD 1.9) years	Linear regression AIMplot^f^ algorithm	AIM^g^ metrics automation	ICCs^h^ 0.95 and 0.94 (intrarater and interrater, respectively)	Inter- and intrarater	v2.0
Jungheim et al, 2016 [32]	Germany	15 healthy volunteers 8 men and 7 women Mean 34.9 years	Logistic regression and sequence labeling	Automated calculation of UES^i^ contraction restitution time	Expert comparable values (restitution time of 11.16 ±5.7s and 10.04 ±5.74s (experts), compared to model-generated values from 8.91 ±3.71s to 10.87 ±4.68s)	Expert comparison	v2.0
Jell et al, 2020 [40]	Germany	15 HRM^j^ for training 25 HRM for validation	Supervised machine learning for automated swallow detection and classification	Automated swallow detection or classification	Accuracy: 97.7% Sensitivity: 89.7% Specificity: 83.2%	Internal validation only	Unspecified
Czako et al, 2021 [41]	Romania	2437 images	InceptionV3 (Google LLC) CNN^k^ for transfer learning	For probe positioning IRP^l^ classification	Accuracy: 97% F1-score >84%	Internal validation only	v2.0
Kou et al, 2021 [33]	United States	2161 HRM studies 32,415 swallows	Variational autoencoder (unsupervised)	Pattern clustering Motility phenotypes	3 distinct clusters in HRM amenable to machine learning classification (linear discriminant)	Internal validation only	v2.0
Kou et al, 2022 [34]	United States	1741 HRM studies 26,115 swallows	LSTM^m^ deep learning	Swallow type classification Peristalsis classification	Swallow type accuracy: 83% Classification of peristalsis accuracy: 88%	Internal validation only	v3.0
Wang et al, 2021 [35]	China	229 esophageal motility cases 229 individuals	3D CNN (Conv3D; Google LLC) Bidirectional convolutional LSTM (BiConvLSTM; Google LLC)	Motility tracing Function mapping	Accuracy: 91.32% Sensitivity: 90.5% Specificity: 95.87%	Internal validation only	v3.0
Kou et al, 2022 [42]	United States	1741 HRM studies	CNNs Extreme gradient boosting Artificial neural network	HRM diagnosis automation	Swallow-type accuracy: 88% Pressurization: 93% Study-level: 81% (top-1), 92% (top-2)	Internal validation only	v3.0
Surdea-Blaga et al, 2022 [36]	Romania	192 HRM studies (patients) 2614 images (1079 IRP, 1535 swallow pattern images)	InceptionV3 for the classification of the IRP DenseNet201 for 5 different classes of swallowing disorders	HRM diagnosis Clouse plot analysis	Top-1 accuracy: 86% F1-score: 86%	Internal validation only	v3.0
Popa et al, 2022 [43]	Romania	1570 images	Inception V3 CNN for transfer learning	HRM diagnosis	Accuracy: 94% Precision: 94% Recall: 93%	Internal validation only	v3.0
Rafieivand et al, 2023 [37]	Iran	67 patients	Graph neural networks Fuzzy classifier	Multi-class esophageal motility disorders diagnosis Decision support	Accuracy: 78.03% (single swallow) Accuracy: 92.54% (patient level)	Internal validation only	v3.0
Zifan et al, 2023 [38]	United States	30 healthy participants 30 patients with functional dysphagia	Multiple models (support vector machines, random forest, k-nearest neighbors, and logistic regression)	Automatic classification of functional dysphagia	Accuracy: 91.7% Precision: 92.86% Logistic regression produced the best results	Internal validation only	v4.0
Zifan et al, 2024 [44]	United States	30 healthy participants 30 patients with functional dysphagia	Ensemble methods (gradient boost, support vector machines, and logit boost)	Functional dysphagia versus controls classification	AUROC: 0.95	Internal validation only	v4.0
Lankarani et al, 2024 [39]	Iran	43 dysphagia patients (suspicious achalasia)	Artificial neural network	To compare the findings on HRM and swallowing sounds	Accuracy: 97%	Internal validation only	v4.0
Popa et al, 2024 [23]	Romania	926 images	CNN ensemble (LLM^n^‑assisted)	Esophageal motility disorder diagnosis	Precision: 89% Accuracy: 88% Recall: 88% F1-score: 88.5%	Internal validation only	v3.0
Wu et al, 2025 [45]	China	2315 swallowing samples	Multi-model CNN attention ensemble	Esophageal motility disorder diagnosis	Accuracy: 98.48%	Internal validation only	v4.0

^a^Characteristics and outcomes of 17 included studies evaluating artificial intelligence for high-resolution manometry interpretation (2013-2025). Studies encompassed 4588 patients from 6 countries (United States, Romania, Germany, Iran, China, and multicenter European studies) with sample sizes ranging from 15 to 2161 participants. Table 1 presents: study design (retrospective, prospective, or validation studies), patient population characteristics, artificial intelligence methodology used (traditional machine learning vs deep learning approaches), specific diagnostic tasks (eg, Chicago Classification diagnosis, integrated relaxation pressure classification, and swallow type identification), reference standards used for model training or validation, diagnostic performance metrics (accuracy, sensitivity, specificity, and area under the receiver operating characteristic curve), and key findings.

^b^AI: artificial intelligence.

^c^MBSImP: Modified Barium Swallow Impairment Profile.

^d^AUROC: area under the receiver operating characteristic curve.

^e^GERD: gastroesophageal reflux disease.

^f^AIMplot: automated impedance manometry analysis.

^g^AIM: automated impedance manometry.

^h^ICC: intraclass correlation coefficient.

^i^UES: upper esophageal sphincter.

^j^HRM: high-resolution manometry.

^k^CNN: convolutional neural network.

^l^IRP: integrated relaxation pressure.

^m^LSTM: long short-term memory.

^n^LLM: large language model.

### Time Trend of AI Application in HRM Interpretation

The application of AI to HRM interpretation has shown continuous evolution since 2013. Early pioneers such as Hoffman et al (2013) [30] applied artificial neural networks to pharyngeal HRM classification, achieving 86.5%-94% accuracy with 335 swallows. During this initial period (2013-2016), researchers focused primarily on automating specific parameter measurements. Rohof et al (2014) [31] created the automated impedance manometry analysis automated analysis system with excellent reproducibility (intraclass correlation coefficient: 0.94-0.95), and Jungheim et al (2016) [32] applied machine learning to calculate upper esophageal sphincter restitution times.

A methodological shift occurred around 2018 when researchers began adopting deep learning approaches. Jell et al (2020) [40] achieved 97.7% accuracy in automated swallow detection using supervised machine learning. The period from 2020-2022 saw widespread adoption of CNNs. Czako et al (2021) [41] achieved 97% accuracy for integrated relaxation pressure (IRP) classification using InceptionV3 (Google LLC) CNN with 2437 images. Kou et al (2021) [33] developed both an unsupervised variational autoencoder analyzing 32,415 swallows from 2161 patients and a supervised long short-term memory network achieving 83% accuracy [34]. Wang et al (2021) [35] implemented temporal modeling with Bidirectional Convolutional long short-term memory networks, reaching 91.32% overall accuracy. Romanian researchers, including Surdea-Blaga et al (2022) [36] and Popa et al (2022) [43], achieved 86% and 94% accuracy, respectively, for Chicago Classification automation.

Recent studies from 2023 onwards have explored increasingly sophisticated and diverse approaches. Zifan et al (2023) [38] used shallow machine learning approaches, including logistic regression, random forests, and k-nearest neighbors, to analyze distension-contraction patterns in 60 patients with functional dysphagia, achieving 91.7% accuracy with logistic regression for proximal segments and 90.5% with random forests for distal segments. Rafieivand et al (2023) [37] developed a fuzzy framework with graphical neural network interpretation, achieving 78% single-swallow accuracy but 92.54% patient-level accuracy in 67 patients. Zifan et al (2024) [44] further refined their approach using support vector machines to analyze distension-contraction plots, achieving an AUROC of 0.95 in 60 patients. Lankarani et al (2024) [39] pioneered noninvasive acoustic analysis combined with AI, achieving 97% accuracy for IRP prediction in 43 patients. Most recently, studies have incorporated large language models, with Popa et al (2024) [23] integrating Gemini with deep learning, while Wu et al [45] (2025) developed mixed attention ensemble approaches (Table 1).

### Diagnostic Accuracy Across Studies

Overall diagnostic accuracies ranged from 78% to 97% across the 17 included studies. The highest accuracies were achieved for specific applications: IRP classification (97%) [41], acoustic IRP prediction (97%) [39], and swallow detection (97.7%) [40]. For Chicago Classification automation, accuracy varied from 86% to >93% [36,43]. Functional dysphagia studies demonstrated segment-specific performance differences, with Rafieivand et al [37] highlighting the importance of patient-level versus swallow-level accuracy (92.54% vs 78%).

Notably, none of the studies provided detailed performance metrics for individual Chicago Classification categories, such as achalasia subtypes or specific motility disorders. This absence of disorder-specific sensitivity and specificity data limits understanding of AI performance across the full spectrum of esophageal pathology and represents a critical gap for clinical implementation (Table 1).

### Methodological Quality

QUADAS-2 assessment revealed variable methodological quality across the 17 included studies (Table 2). For the patient selection domain, no studies demonstrated low risk of bias, with 14 (82%) studies showing unclear risk primarily due to unreported sampling methods, and 3 (18%) studies showing high risk: Hoffman et al [30] included only disordered cohorts without healthy controls, Jungheim et al [32] tested only healthy volunteers limiting representativeness, and Lankarani et al [39] had a small specialized cohort.

**Table 2:** QUADAS-2^a^ methodology quality assessment for included studies^b^.

Study and year	Patient selection	Index test	Reference standard	Flow and timing
Hoffman et al, 2013 [30]	H^c^: no healthy controls	L^d^: clear prespecified threshold	L: expert manual standard method	L: complete data, no losses
Rohof et al, 2014 [31]	U^e^: convenience sample; representativeness unknown	U: calibrated on the same dataset, raising overfitting concerns	U: reproducibility focus, not diagnostic	L: complete data, no losses
Jungheim et al, 2016 [32]	H: healthy only; not representative	U: small n=15, overfit concern	L: reference standard measurements (eg, UES^f^ metrics) and experienced assessors	L: all volunteer data used
Jell et al, 2020 [40]	U: sampling method not reported	L: supervised machine learning clear model	L: expert annotation	L: all data included
Czako et al, 2021 [41]	U: sampling method not reported	L: InceptionV3 (Google LLC) with held-out test	L: expert Chicago‑consistent labels	U: 8 patients excluded, and completeness uncertain
Kou et al, 2021 [33]	U: unclear enrollment method	L: variational autoencoder	H: no validated reference standard	L: all data included
Kou et al, 2022 [34]	U: unclear enrollment method	L: separate test set; blinded automated inference	L: expert Chicago‑consistent labels	L: all data included
Wang et al, 2021 [35]	U: unclear enrollment method	L: train, validation, or test separation	L: expert Chicago‑consistent labels	L: all data included
Kou et al, 2022 [42]	U: unclear enrollment method	L: independent test cohort; rule-based aggregation of swallow‑level models	L: expert Chicago‑consistent labels	L: all data included
Surdea-Blaga et al, 2022 [36]	U: no explicit enrollment stated	L: CNNs^g^ with hold‑out evaluation	L: expert Chicago‑consistent labels	L: all data included
Popa et al, 2022 [43]	U: spectrum bias	L: CNN with internal split	L: expert Chicago‑consistent labels	H: excluded indeterminate cases
Rafieivand et al, 2023 [37]	U: single‑center, small n; sampling not described	L: composite (graph + fuzzy) model	L: expert Chicago‑consistent labels	L: all data included
Zifan et al, 2023 [38]	U: unclear enrollment method	L: multiple machine learning models with cross-validation	U: details of reference adjudication limited	L: all data included
Zifan et al, 2024 [44]	U: unclear enrollment method	L: multiple machine learning models with cross-validation	U: details of reference adjudication limited	L: all data included
Lankarani et al, 2024 [39]	H: small, specialized cohort	L: artificial neural network model	L: expert Chicago‑consistent labels	L: all data included
Popa et al, 2024 [23]	U: unclear enrollment method	L: LLM^h^‑assisted pipeline	L: expert Chicago‑consistent labels	L: all data included
Wu et al, 2025 [45]	U: unclear enrollment method	L: ensemble with cross-validation or hold-out	L: expert Chicago‑consistent labels	L: all data included

^a^QUADAS-2: Quality Assessment of Diagnostic Accuracy Studies-2.

^b^Quality Assessment of Diagnostic Accuracy Studies-2 evaluation of methodological quality and risk of bias for 17 included artificial intelligence studies in high-resolution manometry (2013-2025). Assessment evaluated four domains: (1) patient selection—risk of bias from inappropriate patient selection, exclusions, or case-control design; (2) index test—risk of bias from artificial intelligence model training or validation procedures and threshold determination; (3) reference standard—risk of bias from expert interpretation methods and blinding; and (4) flow and timing—risk of bias from incomplete data or variable intervals between index test and reference standard. Each domain was rated as low risk (L), high risk (H), or unclear risk (U) of bias. Applicability concerns assessed whether study design, patient population, artificial intelligence methodology, or reference standards differed from the review question. The table demonstrates predominant unclear risk in patient selection (14/17, 82% of studies) due to inadequate reporting of recruitment methods, while the index test domain showed the strongest methodological rigor (88% low risk).

^c^H: high risk.

^d^L: low risk.

^e^U: unclear risk.

^f^UES: upper esophageal sphincter.

^g^CNN: convolutional neural network.

^h^LLM: large language model.

The index test domain showed the strongest methodological rigor, with 15 (88%) studies demonstrating low risk of bias through appropriate model training and validation separation. Only 2 (12%) studies showed unclear risk: Rohof et al [31] due to calibration on the same dataset raising overfitting concerns, and Jungheim et al [32] due to the small sample size (n=15), creating uncertainty in algorithm performance.

For the reference standard domain, 14 (82%) studies had a low risk of bias using expert-determined Chicago Classification labels. Further, 3 (18%) studies showed unclear risk: Rohof et al [31] focused on automated metric agreement rather than diagnostic ground truth, and both studies by Zifan et al [38,44] had limited details on reference adjudication. One study by Kou et al [33] showed a high risk as it lacked a validated reference standard for unsupervised clusters.

Flow and timing assessment revealed low risk in 15 (88%) studies, with all patient data included in analyses. One study showed unclear risk (Czako et al [41]) due to the exclusion of 8 patients with probe-placement failure, and 1 study (Popa et al [43]) demonstrated high risk by excluding indeterminate cases from analysis, introducing potential spectrum bias.

The predominance of unclear risk in patient selection highlights a systematic reporting deficiency across the literature, with most studies failing to document recruitment and enrollment methods adequately. This pattern, combined with the complete absence of external validation noted elsewhere, raises concerns about the generalizability and real-world applicability of these AI systems.

### Secondary Findings

None of the 17 included studies performed external validation using datasets from different institutions or periods. All studies relied on internal validation methods, including train-test splits, k-fold cross-validation, or other internal validation approaches. This complete absence of external validation represents a critical limitation in assessing the generalizability of AI models for HRM interpretation. Studies using k-fold cross-validation [35,38,41,44,45] reported more conservative performance estimates compared to simple train-test splits, suggesting potential overfitting in single-split validation approaches.

## Discussion

### Principal Findings

The systematic synthesis of current evidence reveals that AI applications in HRM have demonstrated strong technical performance, with diagnostic accuracies ranging from 78% to 97%, while facing substantial translational challenges. The evolution from traditional machine learning algorithms (86.5%-94% accuracy) to deep learning architectures capable of 97% accuracy for specific tasks represents significant technological progress [30,39,41]. These advances occur within the broader context of AI transformation in gastroenterology, where similar trajectories have been observed in colonoscopy, capsule endoscopy, and inflammatory bowel disease assessment, suggesting that the integration of AI into clinical gastroenterology practice is inevitable rather than speculative [10,11].

The innovation of AI in HRM extends beyond mere automation. These systems represent a major change in how we approach esophageal motility diagnostics [7-10], offering solutions to important clinical needs: the global shortage of motility experts, the need for rapid and consistent interpretation [46], and the potential for telemedicine integration to serve underserved areas [10,11].

The diagnostic accuracy achieved by current AI systems, particularly for IRP classification and automated Chicago Classification, addresses a fundamental limitation of HRM interpretation: interobserver variability. AI systems maintain consistent diagnostic criteria application while human experts demonstrate significant intraobserver variability on repeated assessments. This consistency could enable more reliable phenotyping of esophageal motility disorders, facilitating precision medicine approaches that move beyond categorical diagnoses to individualized pathophysiological assessment. The superior performance of AI in quantitative parameter calculation eliminates measurement variability that has plagued HRM interpretation since its inception [46].

These accuracy levels have important implications for clinical practice. With health care systems facing increasing pressure to reduce costs while improving outcomes, AI-enabled HRM interpretation could decrease repeat procedures and reduce unnecessary testing costs [47,48]. Moreover, the consistent application of diagnostic criteria could reduce misdiagnosis-related treatment failures that currently affect a considerable number of patients with esophageal motility disorders [3,46].

However, the apparent success of AI systems must be contextualized within significant methodological limitations identified through quality assessment. Most critically, no studies demonstrated low risk of bias in patient selection, with 82% (14/17) showing unclear risk due to unreported sampling methods and 18% (n=3) showing high risk due to biased cohort selection [30,32,39]. This systematic deficiency in documenting recruitment and enrollment methods raises fundamental questions about the representativeness of training datasets. The complete absence of external validation across all 17 studies compounds these concerns about generalizability. Internal validation consistently overestimates model performance, and the lack of testing on datasets from different institutions, HRM systems, or patient populations means we have no evidence of real-world performance [10].

The complete absence of prospective clinical trials represents the most critical barrier to clinical translation. While retrospective studies demonstrate technical feasibility with accuracies of 78%-97%, these controlled environments fail to capture the complexities of real-world clinical practice. Prospective trials are essential to evaluate: (1) how AI systems perform with real-time data acquisition variability, (2) whether AI recommendations alter clinical decision-making, (3) patient outcomes following AI-guided treatment, and (4) integration challenges within existing clinical workflows. Without such evidence, even the most accurate AI models remain research tools rather than clinical instruments [9-11].

The evolution through distinct phases of AI development in HRM mirrors broader trends in medical AI but also reveals unique challenges specific to esophageal motility assessment. The transition from traditional machine learning to deep learning approaches yielded substantial performance improvements, yet the “black box” nature of deep learning models poses particular challenges in a field where pathophysiological understanding drives therapeutic decision-making [49]. Clinicians require not just diagnostic labels but mechanistic insights that inform treatment selection between medical therapy, endoscopic intervention, or surgical management. The development of explainable AI models that provide interpretable features and confidence metrics represents a critical priority for clinical acceptance [11]. Recent advances in attention mechanisms and gradient-based visualization techniques, as demonstrated in the Popa et al [23] study using LIME (Local Interpretable Model-Agnostic Explanations), offer promising approaches for making AI decision-making transparent and clinically meaningful.

The integration of multiple diagnostic modalities through AI platforms addresses a longstanding limitation of isolated HRM interpretation. The combination of manometric, impedance, and complementary data provides a more comprehensive assessment of esophageal function than any single modality alone [50]. AI systems excel at synthesizing these complex, multidimensional datasets, potentially revealing pathophysiological patterns invisible to conventional analysis. The Zifan et al (2023 [38] and 2024 [44]) work on distension-contraction plots illustrates how AI can extract diagnostic value from data presentations that challenge human interpretation. This capability becomes particularly relevant with the Chicago Classification version 4.0 emphasis on provocative testing and positional changes, which generate substantially more data requiring integration and interpretation [3].

The absence of disorder-specific performance metrics across all 17 studies severely limits clinical applicability. While overall accuracy appears promising (86%-97%), clinicians need to know how AI performs for specific conditions: distinguishing achalasia subtypes (critical for treatment selection), detecting subtle ineffective esophageal motility (often missed by novices), or identifying rare disorders such as jackhammer esophagus. A system with 95% overall accuracy but poor performance in type II achalasia, for instance, could lead to inappropriate treatment recommendations. Future studies must report sensitivity and specificity for each Chicago Classification category to enable informed clinical decision-making.

Implementation barriers identified across studies reveal a complex interplay of technical, regulatory, clinical, and economic factors. The incompatibility with existing HRM systems reflects the proprietary nature of medical device software and the lack of interoperability standards. The regulatory uncertainty surrounding AI medical devices requires proactive engagement between developers, clinicians, and regulatory agencies to establish appropriate evaluation frameworks [47,48]. Despite these barriers, the economic rationale for AI implementation is strong. High-volume centers could achieve cost-effectiveness through improved workflow efficiency and reduced need for expert consultation [47,48,51], though specific economic analyses are needed to quantify these benefits. The lack of specific reimbursement codes for AI-assisted interpretation creates financial uncertainty that discourages adoption [51]. The potential for AI to enable task-shifting from specialists to general gastroenterologists could address workforce shortages and improve access to motility assessment, particularly in underserved areas.

The ethical implications of AI implementation in HRM diagnostic practice deserve careful consideration [52]. The potential for algorithmic bias, particularly affecting populations underrepresented in training datasets, could exacerbate existing health care disparities. The predominance of studies from North American, European, and select Asian centers raises concerns about applicability to African, Latin American, and other underrepresented populations with different disease phenotypes and genetic backgrounds [52]. Development of quality assurance programs that monitor AI performance and identify edge cases requiring human review will be essential for maintaining patient safety.

Moving from laboratory validation to clinical implementation requires addressing multiple translational gaps simultaneously. First, prospective multicenter trials must demonstrate that AI systems maintain performance across diverse patient populations, HRM equipment, and clinical settings. Second, health economic analyses must quantify whether efficiency gains justify implementation costs—a critical requirement for hospital administrator buy-in and insurance coverage. Third, regulatory pathways need clarification: Should AI-HRM systems be classified as clinical decision support tools or diagnostic devices? Each classification carries different validation requirements and liability considerations. Finally, implementation science research must address workflow integration, user training requirements, and change management strategies to ensure successful adoption [53].

Future priorities must focus on multicenter validation studies, development of explainable AI models, integration with evolving diagnostic frameworks, and systematic addressing of regulatory and economic barriers. The ultimate success of AI in HRM will depend not on technological sophistication alone but on thoughtful integration that preserves clinical judgment while enhancing diagnostic accuracy and efficiency. To achieve clinical translation, the field must transition from technical validation to clinical validation through (1) prospective trials comparing AI-assisted versus standard interpretation on patient outcomes, (2) disorder-specific performance benchmarking across all Chicago Classification categories, (3) cost-effectiveness analyses demonstrating economic value, (4) regulatory sandbox programs allowing controlled real-world testing, and (5) implementation science studies optimizing integration strategies. Until these translational requirements are met, AI in HRM will remain a promising technology awaiting clinical realization.

### Study Limitations

This systematic review has several limitations that should be considered when interpreting the findings. First, the heterogeneity in AI methodologies, patient populations, and outcome definitions precluded meta-analysis, limiting our ability to provide pooled estimates of diagnostic accuracy. Second, we excluded non-English language publications, potentially missing relevant studies from non–English speaking countries. Third, the absence of standardized reporting guidelines for AI studies in HRM made quality assessment challenging, particularly regarding technical aspects of model development. Fourth, publication bias could not be formally assessed due to the diversity of study designs. Fifth, the lack of clinical outcome data across all studies prevented assessment of the real-world impact of AI implementation on patient care, treatment decisions, and health care costs. Finally, critical limitations include the complete absence of low-risk patient selection across all studies, the lack of disorder-specific performance metrics for individual Chicago Classification categories, the absence of prospective clinical trials, no cost-effectiveness analyses, and insufficient direct comparisons between AI and human interpreters using standardized metrics. These gaps collectively limit our ability to assess the true clinical utility and implementation readiness of AI systems in HRM interpretation.

### Conclusions

This systematic review provides comprehensive evidence that AI applications in HRM have achieved remarkable technical capabilities while facing substantial challenges in clinical translation. The diagnostic accuracies of 78%-97% demonstrate the potential for AI to standardize and enhance HRM interpretation. However, the complete absence of external validation, systematic deficiencies in patient selection documentation, and lack of clinical outcome studies highlight the critical gap between technological capability and clinical utility. Additionally, the limited reporting of patient demographics across included studies—reflecting the methodological focus of AI development papers—represents an ongoing challenge for assessing generalizability across diverse populations. Future AI validation studies should systematically report demographic characteristics, including age, sex, race or ethnicity, and geographic location, to enable evaluation of algorithmic performance across patient subgroups and identify potential disparities in diagnostic accuracy that could affect equitable clinical implementation.

## Data Availability

All the data are accessible and available upon reasonable request to the corresponding author.

## Appendix

Multimedia Appendix 1 PRISMA checklist.

Multimedia Appendix 2 PRISMA-DTA checklist.

Multimedia Appendix 3 PRISMA-S checklist.

Multimedia Appendix 4 Search execution documentation.

## Abbreviations

AI: artificial intelligence
AUROC: area under the receiver operating characteristic curve
CNN: convolutional neural network
FLIP: Functional Luminal Imaging Probe
HRM: high-resolution esophageal manometry
IRP: integrated relaxation pressure
LIME: Local Interpretable Model-Agnostic Explanations
PRISMA: Preferred Reporting Items for Systematic Reviews and Meta-Analyses
PRISMA-S: Preferred Reporting Items for Systematic Reviews and Meta-Analyses-Search
PROSPERO: International Prospective Register of Systematic Review
QUADAS-2: Quality Assessment of Diagnostic Accuracy Studies-2
