# Supplementary material for: AI in Esophageal Motility Disorders: Systematic Review of High-Resolution Manometry Studies
Source: J Med Internet Res. 2025 Nov 27;27:e85223. doi: 10.2196/85223 (PMC12699254; doi:10.2196/85223)

**Screenshot documentation (25.11.06)**

**1. Pubmed advanced search tab (https://pubmed.ncbi.nlm.nih.gov/advanced/)**


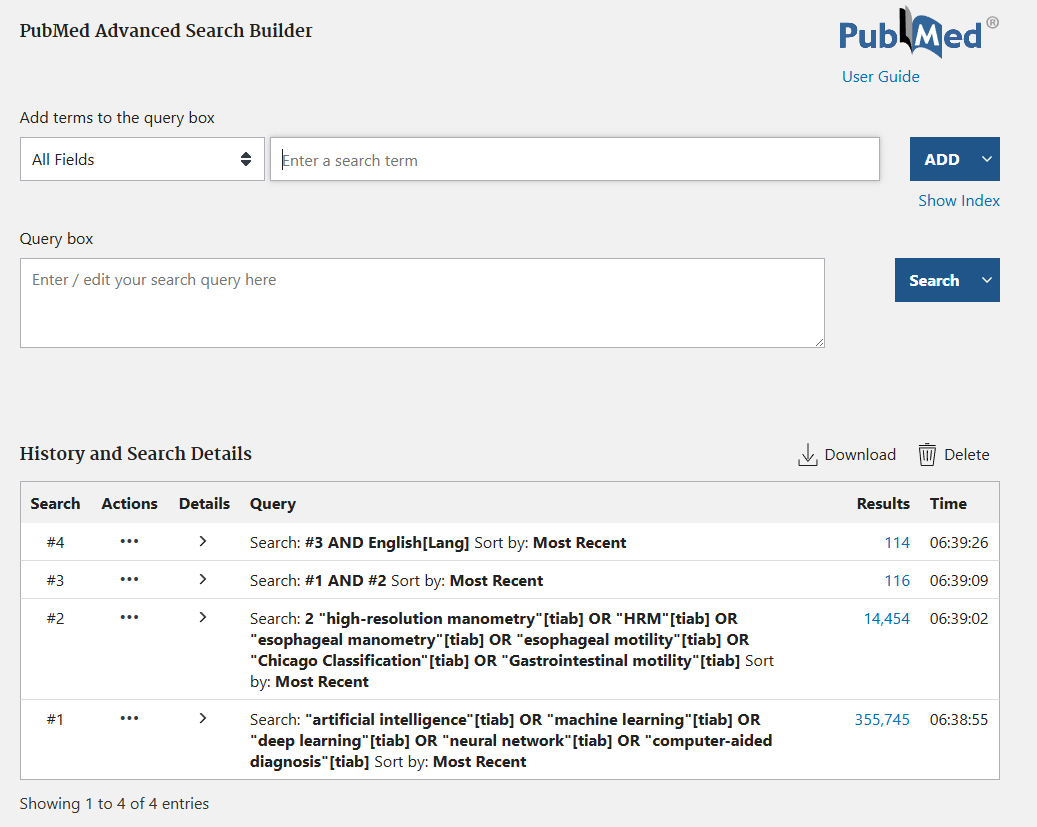


**2. Embase-OVID (**[**https://ovidsp.dc2.ovid.com/ovid-new-a**](https://ovidsp.dc2.ovid.com/ovid-new-a)**)**


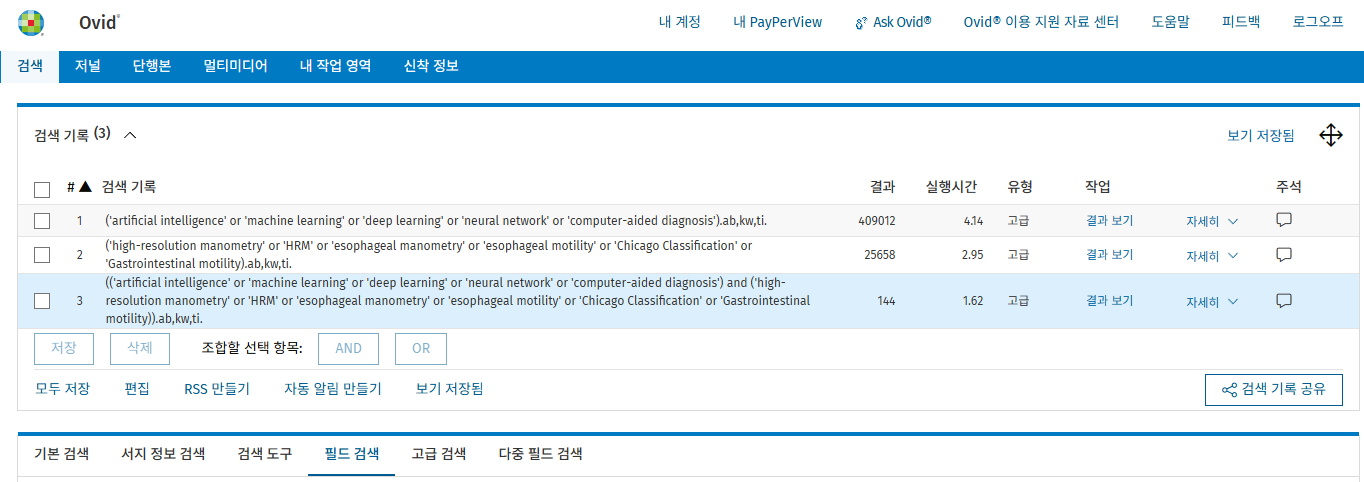


**3. Cochrane library advanced search tab (**[**https://www.cochranelibrary.com/advanced-search/search-manager**](https://www.cochranelibrary.com/advanced-search/search-manager)**)**


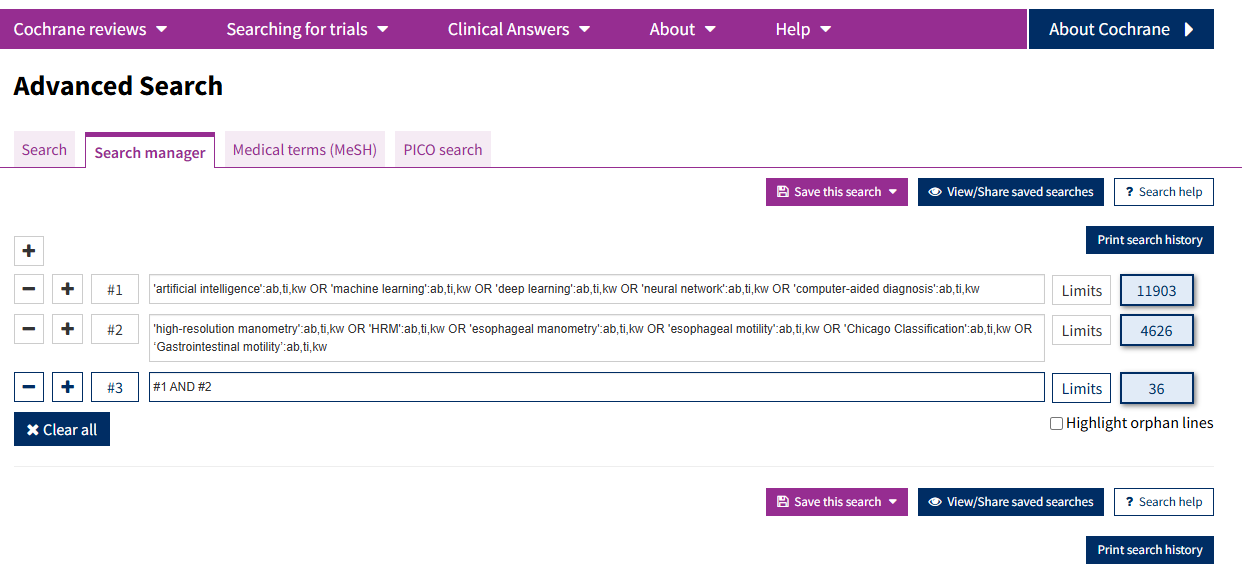


**4. Web of Science advanced search tab (**[**https://www.webofscience.com/wos/woscc/advanced-search**](https://www.webofscience.com/wos/woscc/advanced-search)**)**


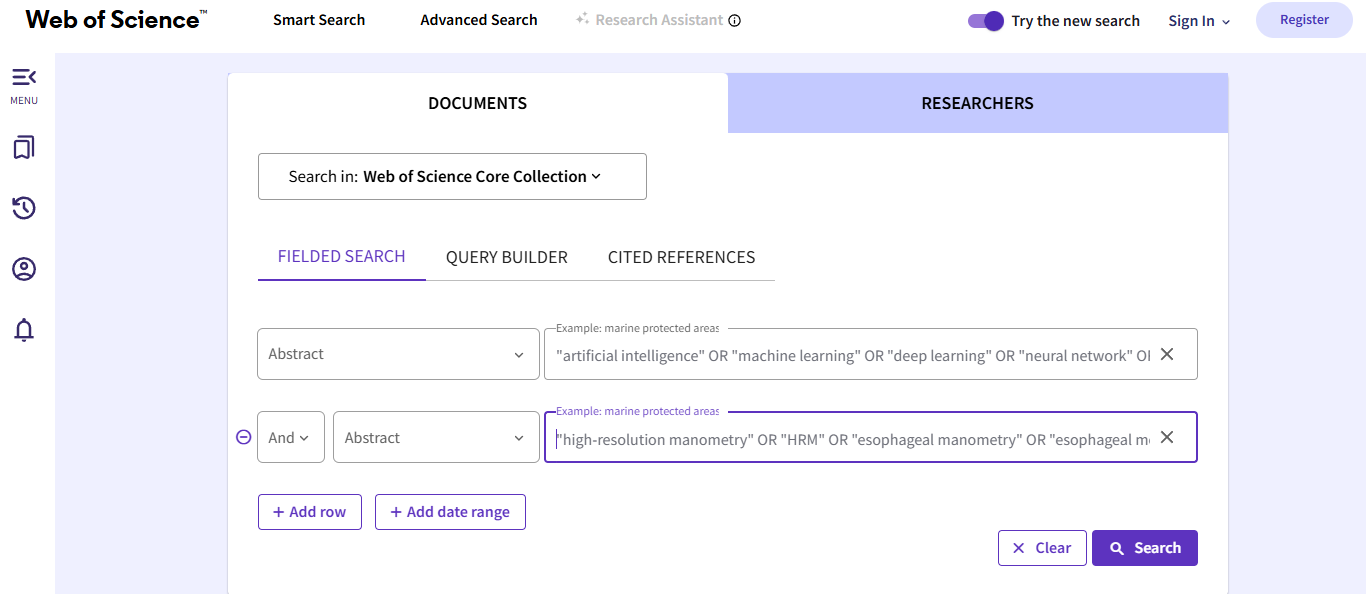


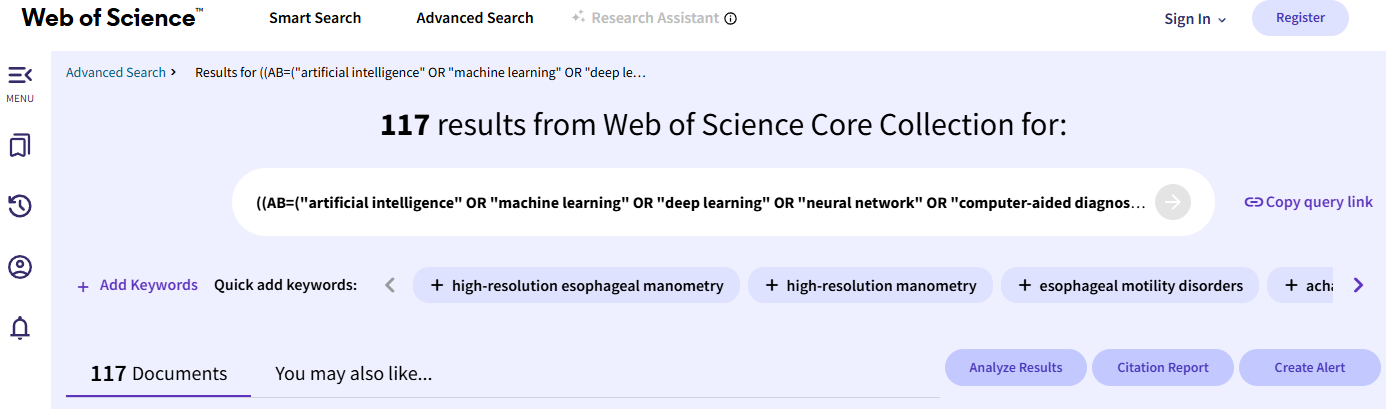


**5. Endnote retrieval snapshot (after duplicated articles removed)**


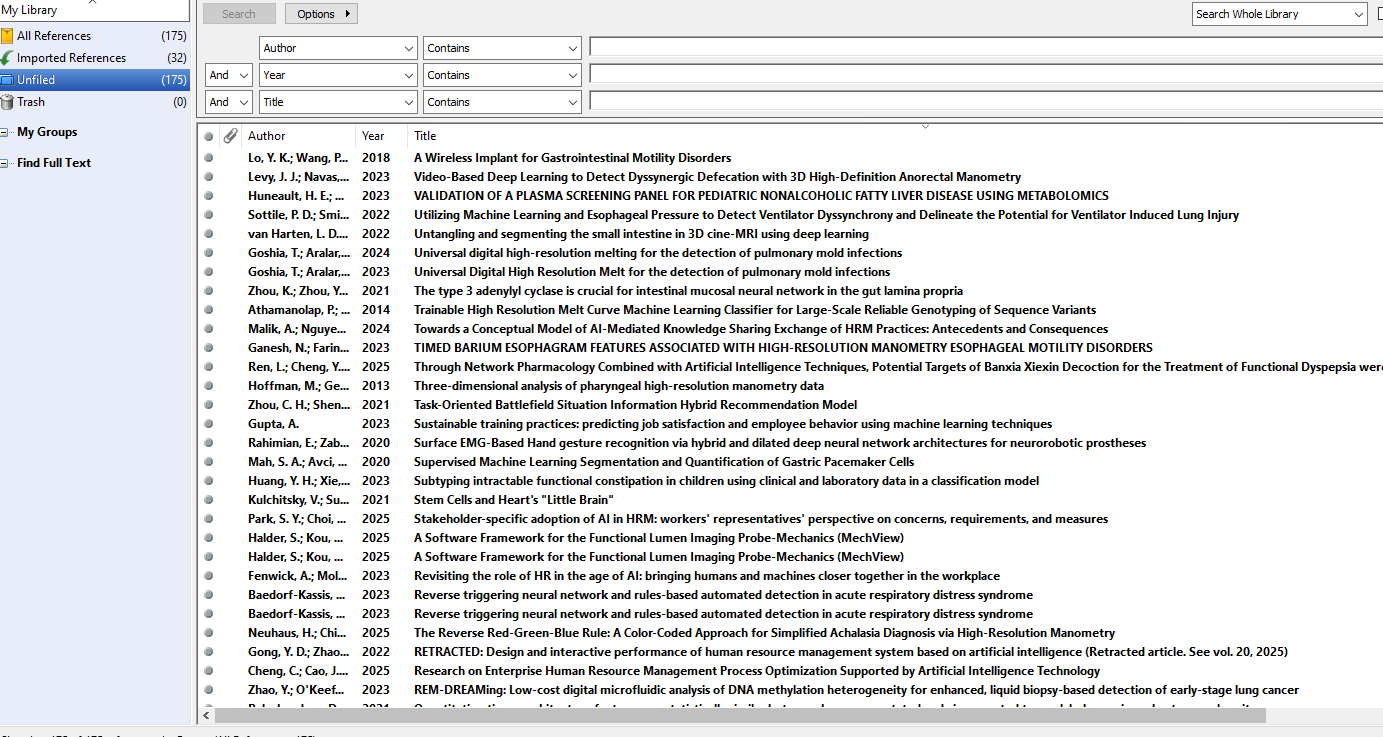

Supplement: Multimedia Appendix 4 [file jmir_v27i1e85223_app4.docx]
